# Efficacy and safety of 5-fluorouracil in infrared monitor guided bleb revision

**DOI:** 10.1186/s12886-021-01843-4

**Published:** 2021-02-08

**Authors:** Rumi Kawashima, Kenji Matsushita, Ryo Kawasaki, Kohji Nishida

**Affiliations:** Department of Ophthalmology, Osaka University Graduate School of Medicine, Room E7, 2-2 Yamadaoka, Suita, Osaka, 565-0871 Japan

**Keywords:** Trabeculectomy, Bleb revision, Bleb knife, Infrared light, 5-fluorouracil

## Abstract

**Purpose:**

Infrared monitor-guided bleb revision (IRGBR), an alternative needling system, visualizes anterior-segment tissues around the bleb not visible during needle revision after trabeculectomy. This study determined the safety and efficiency of 5-fluorouracil (5-FU) as an adjunctive anti-metabolite in IRGBR.

**Methods:**

We retrospectively analyzed 43 consecutive eyes (40 patients; 14 eyes, primary open-angle; 29 eyes, secondary glaucoma) treated with IRGBR for failing filtering blebs. The patients were divided into two groups. The first one had IRGBR without adjunctive 5-FU subconjunctival injection, and the second one had IRGBR with 5-FU. We performed Kaplan-Meier survival analysis using log-rank tests after 2 years of follow-up and Cox proportional hazards regression model to analyze the dependence of the survival time on predictor variables. Two failure criteria were defined as the need for additional surgery for intraocular pressure (IOP) reduction and the IOP at two consecutive follow-up visits based on definition 1, IOP ≧22 mmHg and definition 2, IOP ≧17 mmHg.

**Results:**

Thirty eyes (29 cases) underwent IRGBR with subconjunctival 5-FU injection (group A in the second term) and 13 eyes (11 cases) without 5-FU (group B in the first term). The success rates 24 months after IRGBR were 73.3 and 23.1%, respectively, in groups A and B based on the definition 1 failure and 56.7 and 7.7% based on the definition 2 failure. Complications included transient bleb leaks (group A, 3 eyes; group B, none) and choroidal detachment (group A, 1 eye; group B, none). No use of 5-FU and IOPs ≧10 mmHg 1 week after IRGBR were significant risk factors.

**Conclusions:**

Adjunctive 5-FU in IRGBR achieved a better success rate for failing trabeculectomy blebs.

**Supplementary Information:**

The online version contains supplementary material available at 10.1186/s12886-021-01843-4.

Trabeculectomy is the most popular filtering surgery [1]; however, filtration failure because of subconjunctival scarring and scleral flap fibrotic adhesion can occur often over time and intraocular pressure (IOP) can increase in patients with glaucoma [1]. Bleb needle revision has been performed widely for failing trabeculectomy blebs. We developed the infrared monitor-guided bleb revision (IRGBR) procedure, which uses an infrared monitor to perform the bleb revision with little damage to the scleral flap [2]. 5-Fluorouracil (5-FU), which has anti-metabolic activity and suppresses the wound-healing response, was the first anti-proliferative agent used after trabeculectomy and has been injected subconjunctivally for failing blebs [3, 4]. Needle bleb revision with adjunctive 5-FU is an effective and safe procedure for failing blebs even after the late postoperative period. Previous studies have reported that needle revision with adjunctive 5-FU was a safe procedure and had similar good success rates compared to procedures without 5-FU [5, 6]. The current study compared the 2-year outcomes of IRGBR, our alternative needling system, with and without use of adjunctive 5-FU subconjunctival injections to determine the effect of adjunctive anti-metabolites on this procedure.

## Methods

### Study subjects

The institutional review board of Osaka University Hospital approved this study. We analyzed retrospectively a consecutive case series of patients who had undergone IRGBR for failing filtering blebs between April 2011 and August 2015. We divided the subjects into two groups: subjects who underwent IRGBR without a 5-FU subconjunctival injection between April 2011 and May 2012 and subjects who underwent IRGBR with a 5-FU subconjunctival injection between October 2011 and August 2015, including subjects who had undergone a previous needling procedure.

### Needle bleb revision using IRGBR

One glaucoma specialist (K.M.) performed all procedures using the IRGBR procedure reported previously [2]. After topical anesthesia (4% xylocaine) and topical disinfectant (0.033% polyvinyl alcohol iodine) were applied, patients were placed on the surgical bed and covered with a sterile drape. An eyelid speculum was inserted and the eye was fixed with a corneal traction suture (7–0 silk, Johnson & Johnson, New Brunswick, NJ, USA). We injected 2% xylocaine with epinephrine through a 30-gauge needle into the conjunctiva about 10 mm from the scleral flap. We inserted a bleb knife or a 27-gauge needle into the same site, cut the fibrotic tissues in the bleb, elevated the scleral flap, and inserted the bleb knife into the anterior chamber if possible. In only one case with a tough scleral fibrotic adhesion, we also elevated the flap with vitreous forceps to grab the edge and elevate the flap, because we could observe the flap edge under the infrared monitor. We confirmed bleb formation and decreased IOP followed by suturing of the incision site (10–0 nylon, MANI.INC, Utsunomiya, Tochigi, Japan). We injected 0.1 ml of 5-FU (250 mg/5 ml) into the subconjunctival space. After the bleb revision procedure, we prescribed 1.5% levofloxacin and 0.1% betamethasone sodium phosphate eyedrops 4 times daily for from 1 month to 2 years.

### Data collection

The follow-up data were collected 1 week and 1, 3, 6, 12, 18, and 24 months after IRGBR. The IOP was measured at each visit by Goldmann applanation tonometry. The patients underwent complete ophthalmic examinations to detect bleb revision complications.

### Outcome measures

We assessed the 2-year outcome after IRGBR with 5-FU using two different failure definitions based on the need for another surgery for IOP reduction regardless of IOP-lowering medications and IOP. Initially, failures were defined as the need for another surgery to lower the IOP during the follow-up period. Second, failures were defined as an IOP at two consecutive visits higher than the following criteria: 1, IOP ≧22 mmHg; and 2, IOP ≧17 mmHg. We analyzed the success rates of IRGBR and number of adverse events.

### Statistical analysis

The statistical analyses were performed using JMP Pro 14 software (SAS Institute Inc., Cary, NC, USA) following expert technical advice. The demographic data were analyzed using the Wilcoxon rank test or χ2 test. *P* <  0.05 was considered statistically significant. We compared the success rates for each group using the Kaplan-Meier method and log-rank test. We evaluated the statistical power using the post-hoc power calculator. We analyzed the risk factors for failure of IRGBR using Cox hazard analysis.

## Results

Forty-three eyes of 40 patients were included in the study. Among them, 30 eyes of 29 cases underwent IRGBR with 5-FU subconjunctival injection (group A) and 13 eyes of 11 cases without 5-FU (group B).

### Subject demographic data

The demographic data are summarized in Table 1. Fifteen (51.7%) men and 14 (48.3%) women were in group A, and seven (63.6%) men and four women (36.4%) were in group B. The mean age at the time of the needle bleb revision was 61.0 ± 16.2 in group A and 63.8 ± 13.5 in group B. The rate of primary open-angle glaucoma was 30% in group A and 38.5% in group B. The mean duration between trabeculectomy with mitomycin C (MMC) and IRGBR was 767.8 ± 898.9 days (range, 16–2819) in group A and 890.2 ± 1448.0 days (range, 15–4724) days in group B. The IOP before IRGBR was 28.9 ± 9.3 mmHg in group A and 26.6 ± 9.6 mmHg in group B. The numbers of IOP-lowering medications were 2.5 ± 2.0 in group A and 2.0 ± 2.1 in group B. No significant differences were seen between the two groups.

**Table 1:** Patient demographic data

	With 5-FU Needle Revision Group (*n* = 30)	Without 5-FU Needle Revision Group (*n* = 13)	*P* Value
Sex			0.48*
Men	15 (51.7%)	7 (63.6%)	
Women	14 (48.3%)	4 (36.4%)	
Age at needling (years)	61.0 ± 16.2	63.8 ± 13.5	0.68†
Disease			0.75*
Primary open-angle glaucoma	9 (30%)	5 (38.5%)	
Secondary glaucoma	21 (70%)	8 (61.5%)	
Neovascular glaucoma	2 (6.7%)	0	
Exfoliation glaucoma	6 (20%)	0	
Uveitis	4 (13.3%)	4 (30.8%)	
Others	9 (30%)	4 (30.8%)	
Previous intraocular surgery	1.4 ± 1.33	1.0 ± 1.44	0.28†
Time between previous trabeculectomy and needling (days)	767.8 ± 898.9	890.2 ± 1448.0	0.30†
〜6 months	14 (46.7%)	8 (61.5%)	
6 months 〜 2 years	5 (16.7%)	1 (7.7%)	
2 years 〜	11 (36.7%)	4 (30.8%)	
IOP pre-needling (mmHg)	28.9 ± 9.3	26.6 ± 9.6	0.28†
Score of glaucoma medications pre-needling (point)	2.5 ± 2.0	2.0 ± 2.1	0.38†

*χ^2^ test

†Wilcoxon rank test

5-FU, 5-fluorouracil; IOP, intraocular pressure

### Clinical outcomes of IRGBR

**Fig. 1:**
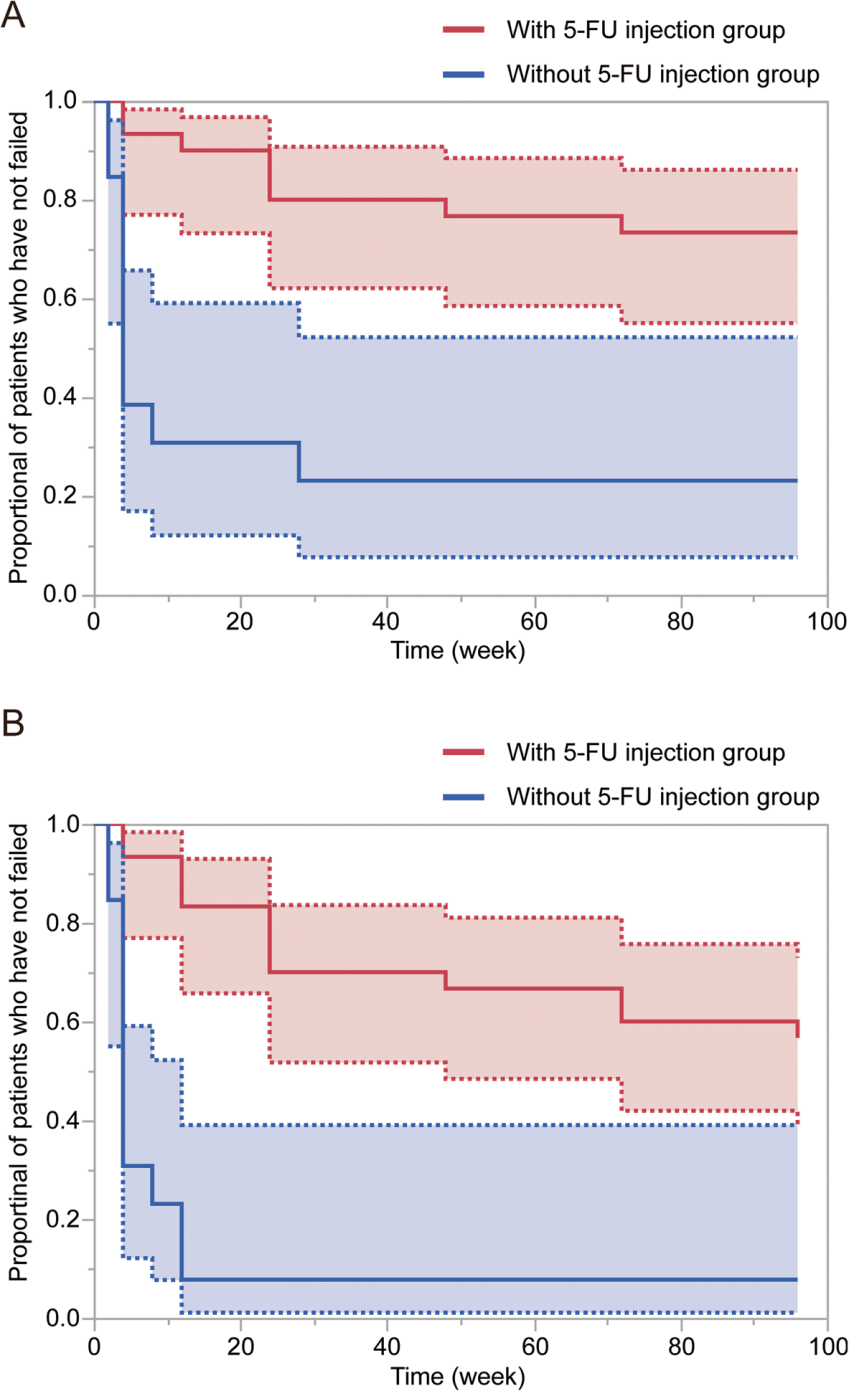
Survival curves after infrared guided bleb revision with and without adjunctive 5-fluorouracil (5-FU) subconjunctival injections. **a**, According to failure definition 1, significant differences are seen between the groups in the cumulative failure rates at 24 months after the procedure (*P* < 0.0001, log-rank test). **b**, According to failure definition 2, significant differences are seen between the cumulative failure rates 24 months after the procedure (*P* < 0.0001, log-rank test). The red line indicates the group in which 5-FU was used. The blue line indicates the group in which 5-FU was not used. The dotted lines indicate the 95% confidence interval

Table 2 shows the success rates of IRGBR, which according to failure definition 1 were 93.3% in group A and 38.5% in group B 1 month after IRGBR; 76.7 and 23.1%, respectively, at 12 months; and 73.3 and 23.1%, respectively, at 24 months. The success rates according to failure definition 2 were 93.3% in group A and 30.8% in group B 1 month after IRGBR; 66.7 and 7.7%, respectively, at 12 months; and 56.7 and 7.7%, respectively, at 24 months (Fig. 1). At 24 months after IRGBR, the difference in the success rates of the two groups reached significance based on both definitions (*P* <  0.0001 for both comparisons by the log-rank test). We evaluated the statistical power using the post-hoc power calculator. We used the following statistical parameters in in definition 1: number of subjects: group A, 30; group B, 13; study incidence: group A, 73.3%; group B, 23.1%; and alpha: 0.05. The post-hoc power was 89.9%. We then evaluated the power in definition 2 with the following statistical parameters: number of subjects: group A, 30; group B, 13; study incidence: group A, 56.7%; group B, 7.7%; and alpha: 0.05. The post-hoc power was 92.6%.

**Table 2:** Success rate of IRGBR

	*n*	With 5-FU Needle Revision Group	n	Without 5-FU Needle Revision Group	*P* Value
Failure definition 1	30		13		
1 month	28	93.3	5	38.5	
3 months	27	90.0	4	30.8	
6 months	24	80.0	4	30.8	
12 months	23	76.7	3	23.1	
24 months	22	73.3	3	23.1	**< 0.0001**
Failure definition 2	30		13		
1 month	28	93.3	4	30.8	
3 months	25	83.3	1	7.7	
6 months	21	70.0	1	7.7	
12 months	20	66.7	1	7.7	
24 months	17	56.7	1	7.7	**< 0.0001**

*P* value estimated using the log-rank test

*5-FU* 5-fluorouracil, *IRGBR* infrared-guided bleb revision

### IRGBR safety

During the observation period, no major complications developed in group B. In group A, three (10%) patients had transient bleb leaks and one (3.3%) had hypotony with a choroidal detachment after IRGBR (Table 3). Among those with a bleb leak, one patient needed additional sutures and one underwent needle revision to reduce the bleb pressure. The choroidal detachment recovered with conservative treatment.

**Table 3:** Complications after IRGBR

	*N* (%)
With 5-FU	4 (13.3)
Bleb leak	3 (10.0)
Hypotony	1 (3.3)
Without 5-FU	0

*IRGBR* infrared-guided bleb revision, *5-FU* 5-fluorouracil

### Risk factors for failure of IRGBR

We analyzed the risk factors for time to failure of IRGBR using a Cox hazard model. Table 4 shows the result of the univariable Cox proportional hazard ratio and multivariable Cox proportional hazards regression using nine variables, i.e., age, sex, glaucoma type, 5-FU, number of previous intraocular surgeries, number of IOP-lowering medications pre-IRGBR, IOP pre-IRGBR, IOP 1 week after IRGBR, and time between trabeculectomy and IRGBR. Using failure definition 1, 5-FU and IOP 1 week after IRGBR were significant risk factors for IRGBR failure according to a univariable analysis (odds ratios [ORs], 5.07, 4.63; 95% confidence intervals [CIs], 1.95–13.2, 1.51–14.2; *P* = 0.0009, *P* = 0.007) and 5-FU, IOP 1 week, and age after IRGBR were significant in a multivariable analysis (ORs, 4.90, 5.54, 3.55; 95% CIs, 1.37–17.5, 1.31–23.4, 1.06–11.9; *P* = 0.01, *P* = 0.02, *P* = 0.04, respectively). Using definition 2, 5-FU and IOP 1 week after IRGBR were significant risk factors according to univariable analysis (ORs, 6.29, 3.19; 95% CIs, 2.65–15.0, 1.36–7.50; *P* <  0.0001, *P* = 0.008) and 5-FU, IOP 1 week after IRGBR, and disease were significant in multivariable analysis (ORs, 6.84, 3.99, 3.43; 95% CIs, 2.28–20.5, 1.30–12.3, 1.18–9.95; *P* = 0.0006, *P* = 0.02, and *P* = 0.02, respectively). These results indicated that no use of 5-FU and IOP ≧ 10 mmHg 1 week after IRGBR were significant risk factors for failure of IRGBR.

**Table 4:** Risk factor analysis for IRGBR failure

	Failure Definition 1				Failure Definition 2			
	Univariate		Multivariate		Univariate		Multivariate	
	HR (95% CI)	*P* Value	HR (95% CI)	*P* Value	HR (95% CI)	*P* Value	HR (95% CI)	*P* Value
Age (years)								
≧ 65	Reference		Reference		Reference		Reference	
<65	1.78 (0.67–4.75)	0.25	**3.55 (1.06–11.9)**	**0.04**	0.70 (0.32–1.54)	0.38	0.73 (0.30–1.80)	0.50
Sex								
Men	Reference		Reference		Reference		Reference	
Women	1.20 (0.48–3.03)	0.70	1.56 (0.48–5.15)	0.45	1.10 (0.50–2.40)	0.82	1.41 (0.53–3.78)	0.49
Glaucoma								
SOAG	1.10 (0.41–2.94)	0.85	1.13 (0.33–3.92)	0.85	1.45 (0.60–3.47)	0.41	**3.43 (1.18–9.95)**	**0.02**
POAG	Reference		Reference		Reference		Reference	
5-FU								
Yes	Reference		Reference		Reference		Reference	
No	**5.07 (1.95–13.2)**	**0.0009**	**4.90 (1.37–17.5)**	**0.01**	**6.29 (2.65–15.0)**	**< 0.0001**	**6.84 (2.28–20.5)**	**0.0006**
No. previous surgeries								
≦ 1	Reference		Reference		Reference		Reference	
≧ 2	0.95 (0.34–2.67)	0.93	2.29 (0.50–10.5)	0.29	0.93 (0.39–2.23)	0.87	1.47 (0.45–4.76)	0.53
No. IOP-lowering medications pre-needling								
≦ 2	Reference		Reference		Reference		Reference	
≧ 3	1.20 (0.47–3.07)	0.70	1.21 (0.32–4.53)	0.78	1.10 (0.44–3.30)	0.85	1.38 (0.48–3.99)	0.55
IOP pre-needling (mmHg)								
≧ 25	Reference		Reference		Reference		Reference	
<25	1.52 (0.60–3.82)	0.38	1.73 (0.51–5.86)	0.38	1.52 (0.69–3.34)	0.30	1.97 (0.72–5.41)	0.19
IOP 1 week after needling (mmHg)								
<10	Reference		Reference		Reference		Reference	
≧ 10	**4.63 (1.51–14.2)**	**0.007**	**5.54 (1.31–23.4)**	**0.02**	**3.19 (1.36–7.50)**	**0.008**	**3.99 (1.30–12.3)**	**0.02**
Time between surgery and needling (years)								
<1	Reference		Reference		Reference		Reference	
≧ 1	0.61 (0.23–1.63)	0.33	0.93 (0.27–3.20)	0.91	0.66 (0.29–1.49)	0.31	0.87 (0.31–2.40)	0.79

*P* values estimated using Cox proportional hazard model

*HR* hazard ratio, *CI* confidence interval, *5-FU* 5-fluorouracil, *IOP* intraocular pressure, *IRGBR* infrared-guided bleb revision, *POAG* primary open-angle glaucoma; SOAG, secondary open-angle glaucoma

## Discussion

This mid-term study showed that the efficacy of 5-FU subconjunctival injection of IRGBR after trabeculectomy was better than that reported previously. The complication rate was lower and no severe complications developed. Broadway et al. [7]. reported a 75% success rate at 1 year and 52% at 3 years after bleb needle revision with 5-FU (IOP success defined as ≦21 mmHg and a 30% reduction with additional glaucoma eyedrops). In addition, Rotchford and King [8] reported 54.3% at 1 year and 45.7% at 2 years (IOP success defined as ≦21 mmHg and under the target IOP with additional glaucoma eyedrops), Kapasi and Birt [9] reported 35.1% at 2 years (IOP success defined as under the target IOP with additional glaucoma eyedrops, laser treatment, or repeated bleb needle revision), Zheng et al. [10] reported 42% at 1 year and 2 years and 27% at 4 years (IOP success defined as ≦20 mmHg with additional glaucoma eyedrops), and Kim et al. [6] reported 44.6 and 32.9% at 2 years (IOP success defined as ≦21 mmHg or ≦85% of the preoperative IOP and ≦18 mmHg or ≦80% of the preoperative IOP). In the current study, we showed that IRGBR made easy reconstruction of the functional bleb possible by detaching the fibrotic tissue adhesion noninvasively and by penetrating the outflow pathway into the anterior chamber safely even if it adhered tightly to the bleb and the scleral flap. The success rates of IRGBR without 5-FU were significantly lower than with 5-FU in our data, 23.1 and 73.3% according to failure definition 1, and 7.7 and 56.7% according to failure definition 2 at 2 years after the procedure. The low success rate of IRGBR without 5-FU might have resulted from the high rate of secondary glaucoma.

Regarding the risk factors for failure of needle revision, our data showed that no use of 5-FU use and IOP ≧10 mmHg 1 week after IRGBR were the significant risk factors. A high pre-needling IOP, no MMC use during the previous filtration surgery, high IOP immediately after needling revision, bleb morphology, and a greater total number of needle revisions have been documented as risk factors for failure of needle revision with 5-FU [6, 8, 11]. This result might show that adjunctive 5-FU injection after needle revision is effective for failing blebs in eyes with secondary glaucoma.

The current retrospective study had some limitations included unmasked controls, a small sample size, and some patients with short follow-up times. The number of group B cases in which 5-FU was not injected was less than in group A in which 5-FU was injected because this was a consecutive retrospective study. However, fortunately, post-hoc analysis showed that our sample size was sufficiently large. The current study was intended to be a pilot study to report this novel modification of a needling approach with the use of 5-FU injection in IRGBR. We will investigate the efficacy of IRGBR with 5-FU in a larger future study. The success rate could be affected by the learning curve associated with a new procedure. However, using the new procedure, we can observe the surgical field with both a stereotyped surgical microscope and a new infrared monitor. A learning curve is not relevant, because we can revert to the original method immediately when we experience difficulties. In fact, an additional analysis indicated that the time of surgery did not pose a major problem (Additional Figure).

In conclusion, IRGBR is a safe and effective procedure. In addition, use of adjunctive 5-FU after IRGBR was an important factor in the success of needle revision of secondary glaucoma in the mid-term.

## Supplementary Information

**Additional file 1: Figure S1**. Survival curves after infrared guided bleb revision depending on the date of the needling.

## Data Availability

The datasets used and analysed during the current study are available from the corresponding author on reasonable request.

## Abbreviations

IRGBR: Infrared monitor-guided bleb revision
5-FU: 5-fluorouracil
IOP: Intraocular pressure
MMC: Mitomycin C
ORs: Odds ratios
CIs: Confidence intervals
