# Supplementary material for: Efficacy and safety of 5-fluorouracil in infrared monitor guided bleb revision
Source: BMC Ophthalmol. 2021 Feb 8;21:75. doi: 10.1186/s12886-021-01843-4 (PMC7869480; doi:10.1186/s12886-021-01843-4)
Supplement: Supplementary file 1 — Additional file 1: Figure S1. Survival curves after infrared guided bleb revision depending on the date of the needling. [file 12886_2021_1843_MOESM1_ESM.docx]

**Supplementary information**

Efficacy and Safety of 5-Fluorouracil in Infrared Monitor Guided Bleb Revision

Rumi Kawashima, MD, PhD, Kenji Matsushita, MD, PhD*, Ryo Kawasaki, MD, PhD, and Kohji Nishida, MD, PhD

From Osaka University Graduate School of Medicine, Department of Ophthalmology, 2-2 Yamadaoka, Suita, Osaka, Japan.

**Additional Figure.**

Survival curves after infrared guided bleb revision depending on the date of the needling.


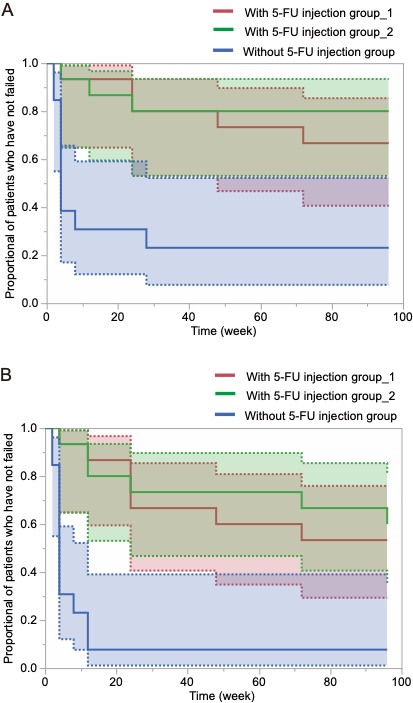


A, According to failure definition 1, significant differences are seen between group 1 with no use of 5-FU and group 2 with use of 5-FU2, and between group 1 with no use of 5-FU and group 1 with use of 5-FU (P=0.001, P=0.002, respectively, by the log-rank test). Group 1 with use of 5-FU and group 2 with use of 5-FU do not differ significantly (P=0.56, by the log-rank test) in the cumulative failure rates at 24 months after the procedure. B, According to failure definition 2, significant differences are seen between group 2 without use of 5-FU and group 2 with use of 5-FU, and group 1 without use of 5-FU and group 1 with use of 5-FU (P<0.0001 for both comparisons, by the log-rank test). Group 1 with use of 5-FU and group 2 with use of 5-FU do not differ significantly (P=0.74, by the log-rank test) in the cumulative failure rates at 24 months after the procedure. The green and red lines indicate the groups in which 5-FU was used. The use of 5-FU injections are divided into 2 groups based on the date of the needling procedure: green, group 1, October 2011 to August 2013 (n=15); red, group 2, November 2013 to Aug 2015 (n=15). The blue lines indicate the groups in which 5-FU was not used: April 2011 to May 2012 (n=13). The dotted lines indicate the 95% confidence intervals.
